# Evaluating Flinders Technology Associates card for transporting bacterial isolates and retrieval of bacterial DNA after various storage conditions

**DOI:** 10.14202/vetworld.2020.2243-2251

**Published:** 2020-10-28

**Authors:** Azhar G. Shalaby, Neveen R. Bakry, Abeer A. E. Mohamed, Ashraf A. Khalil

**Affiliations:** 1 Department of Biotechnology Unit, Reference Laboratory for Veterinary Quality Control on Poultry Production, Animal Health Research Institute, Agricultural Research Centre, Dokki, Giza, Egypt; 2 Department of Epidemiology Unit, Reference Laboratory for Veterinary Quality Control on Poultry Production, Animal Health Research Institute, Agricultural Research Centre, Dokki, Giza, Egypt; 3 Department of Buffalo Diseases, Animal Health Research Institute, Dokki, Giza, Egypt; 4 Institute of Biotechnology and Genetic Engineering, City of Scientific Research and Technology Applications, Borg Elarab, Alexandria, Egypt

**Keywords:** bacteria, colony-forming units, Flinders Technology Associates, nucleic acid, polymerase chain reaction

## Abstract

**Background and Aim:**

Flinders Technology Associates (FTA) cards simplify sample storage, transport, and extraction by reducing cost and time for diagnosis. This study evaluated the FTA suitability for safe transport and storage of Gram-positive and Gram-negative bacterial cells of animal origin on its liquid culture form and from organ impression smears (tissues) under the same routine condition of microbiological laboratory along with detecting their nucleic acid over different storage conditions.

**Materials and Methods:**

Increase in bacterial count from 10^4^ to 10^7^ (colony-forming units/mL) of 78 isolates representing seven bacterial species was applied onto cards. FTA cards were grouped and inoculated by these bacteria and then stored at different conditions of 24-27°C, 4°C, and −20°C for 24 h, for 2 weeks, for 1 and 3 month storage, respectively. Bacteriological examination was done, after which bacterial DNA was identified using specific primers for each bacterial type and detected by polymerase chain reaction (PCR).

**Results:**

The total percentage of recovered bacteria from FTA cards was 66.7% at 24-27°C for 24 h, the detection limit was 100% in Gram-positive species, while it was 57.4% in Gram-negative ones. Regarding viable cell detection from organ impression smears, it was successful under the previous conditions. No live bacterial cells were observed by bacteriological isolation rather than only at 24-27°C for 24 h storage. All bacterial DNA were sufficiently confirmed by the PCR technique at different conditions.

**Conclusion:**

Overall, the FTA card method was observed to be a valid tool for nucleic acid purification for bacteria of animal origin in the form of culture or organ smears regardless of its Gram type and is used for a short time only 24 h for storage and transport of live bacteria specifically Gram-positive type. Moreover, the bacterial nucleic acid was intact after storage in −20°C for 3 months and was PCR amplifiable.

## Introduction

It is desirable for laboratories to maintain bacterial strains in culture for extended time for research, teaching, and quality control purposes. Flinders Technology Associates (FTA) helps in gathering, purification, and storage of genetic material from various biological sources [1]. FTA card simplifies sample storage transport and DNA extraction, thereby reducing the cost and time for diagnosis [2]. The chemicals impregnated in that filter paper have made the samples as non-infectious, therefore minimizing the biohazards, especially during shipment [3]. In addition, FTA card chemical structure enhances cell lysis and DNA binding activity to the surface, in addition to nucleic acid protection against degradation [4]. Moreover, using FTA card is helpful in large field sampling for surveillance, yielding a sufficient amount of nucleic acid that is still stable at 24-27°C for further screening over time of storage at 24-27°C [5].

The hazardous biological agents have a possible risk of infection in animal species and humans, despite that its shipment requires a secured condition for transportation and specific time-temperature circumstances to assure safety handling and satisfactory diagnostic process in laboratory condition [6]. The FTA method is used in areas with limited facility for storage and these cards are also useful for nucleic acid collection from unconventional sources like mosquito and preserve the nucleic acid of viruses for further investigation [7]. FTA cards are useful in forensic studies as its chemical structure saves the samples from deterioration until the final analysis [8]. Different zoonotic infectious bacteria can be transmitted to human through different routes, such as food and water, among others. Furthermore, most pathogens are normally inhabitant in apparently healthy animals and may transmit diseases through products (meat or meat product, poultry meat, or its product milk and eggs) [9]. There are different bacterial agents in various animals and poultry with high zoonotic importance like *Staphylococcus* *aureus*, causing mastitis in cows and buffaloes [10], *Listeria* spp. specially *Listeria* *monocytogenes* [11], and *Mycobacterium tuberculosis* bacilli during their treatment or from animal source [12]. FTA cards served as storage tools for different microbial nucleic acids such as *Salmonella* [13] and *Campylobacter* [14], which could be transmitted through milk products and milk samples from apparently healthy buffaloes and other ruminants [15]. *Escherichia* *coli* is also one of the pathogenic agents in avian species [16] causing calf diarrhea [17], and another infectious agent is *Pseudomonas aeruginosa* [18]. Furthermore, FTA card commercial tools are possible tools for detecting *Pasteurella multocida* DNA in all samples tested and for storage period up to 35 days at 4°C and 37°C [19].

The FTA cards become an excellent transport tool for collection and archiving of samples for diagnosing some poultry viruses and can be widely used for shipment of samples [20]. Therefore, FTA cards have been designed to keep the pure bacterial culture [1] and the nucleic acids of infectious agents directly from tissues by the mean of impression smears allow safety gathering and nucleic acid storage [3]. Furthermore, it was found in different forensic laboratories as required various DNA analysis techniques [21].

FTA cards have been approved as an excellent tool for shipment, collection and diagnosis of some poultry viruses [20]. Therefore, it is considered an easy way to collect samples from different areas without the need of cool chain or safety measures during shipment of hazardous reagents [6]. Furthermore, the FTA cards facilitate the transport of different virological samples in countries where the reverse cold chain is unavailable [22].

Whatman FTA cards were widely used to store different types of samples like tissues and cells. Also, nucleic acid could be stored onto cards and amplified for different molecular analysis [23].

This study evaluated the FTA suitability for safe transport and storage of Gram-positive and Gram-negative bacterial cells of animal origin on its liquid culture form and from organ impression smears (tissues) under the same routine condition of microbiological laboratory along with detecting their nucleic acid over different storage conditions.

## Materials and Methods

### Ethical approval

This study does not require ethical approval. However, Institutional and National guidelines for the care and use of animals were followed according to the guidelines approved by the ethics committee of Animal Health Research institute.

### Samples

Two hundred samples were collected from domestic farms of Giza Governorate from January-2018 to December-2019 from apparently healthy and diseased buffaloes and chickens as follows: 114 samples from buffaloes (93 raw and mastitis buffalo milk, 17 fecal matter, and 4 skin lesion samples) and 86 samples from chicken (liver 16, spleen 7, heart 33, and lungs 30). All samples were collected under full aseptic conditions in separate labeled containers, and then quickly sent to the laboratory in an icebox for bacteriological examination. For closed edematous skin lesions of buffalo samples, disinfection of the surface using 5% tincture iodine was applied.

### Bacteriological examination

Samples were bacteriologically examined according to Quinn *et al*. [24]. One milliliter of each sample was aseptically collected, cultured in 9 mL of different enrichment broths, and also secondary enrichment was streaked onto different agar media (Table-1).

**Table 1:** Types of enrichments for different bacterial species.

Family	Pre-enrichment	Selective enrichment
*Listeriaceae*	Listeria enrichment broth (UVM I), UVM II (Frazer broth	Oxford agar
*Pasteurellaceae*	Nutrient broth	Blood agar
*Pseudomonadaceae*	Nutrient broth	Pseudomonas selective agar with C, N supplement
*Enterobacteriaceae*	Nutrient broth	MacConkey agar, Eosin methylene blue agar (EMB)
*Staphylococcaceae*	Nutrient broth	Baird parker
*Yersiniaceae*	Trypticase soy broth	Yersinia selective agar supplemented with cefsulodin, irgasan, novobiocin (CIN) Yersinia supplement
*Corynebacteriaceae*	Nutrient broth	Blood agar and brain–heart infusion agar plates

### Total bacterial count

One milliliter of each sample was harvested by centrifugation at 13,400*×g* for 5 min, washed, and serially diluted in 0.1% peptone water to yield cell suspensions ranging from 10^0^ to10^8^ colony-forming unit (CFU)/mL. The bacterial population was determined by plating 0.1 mL portion of appropriately diluted culture on duplicate selective agar plates and incubated at 37°C for 48 h. The total colony count per mL of broth culture was then calculated and recorded [25].

### Conventional polymerase chain reaction (PCR) for detecting conserved genes for isolated strains

#### Inoculating FTA cards

Fifty microliters cell suspension was pipetted and spotted onto duplicate FTA card sample area according to the instructions of Whatman [26], ensuring an even distribution. Furthermore, impression smears of tissue organs on FTA cards were obtained. FTA cards were allowed to dry at 24-27°C in a biosafety cabinet Class A II (Thermo Fisher, Germany) for 1-2 h and stored in a dry dark place. The cards were then grouped into four groups as follows: Group (A), the cards were incubated at 24-27°C for 24 h; Group (B), incubation was performed at 4°C for 2 weeks; Group (C), cards were stored at −20°C for 1 month; and Group (D), cards were kept at −20°C for 3 months. The viability of bacteria on FTA cards was tested by embedding paper disc pieces (with a sterile punch) of each bacterial type in phosphate buffer saline (pre-enrichment media) for 24 h at 37°C, then inoculated on relevant selective medium. Cards without bacterial inoculation served as a control.

For detecting DNA from inoculated FTA cards, the cards were punched out using a 2.0 mm Harris Micro Punch (Whatman Inc., Sigma-Aldrich, UK) and transferred into sterile 1.5 mL Eppendorf tubes before adding 200 μ FTA purification reagents. Tubes were mixed and homogenized for 2 min (TissueLyser, LT. Qiagen, Germany) and then incubated at 56°C for 5 min. The purification reagent was discarded and replaced with fresh one to repeat this step one more time. DNA was purified from bacterial isolates inoculated onto cards using QIAamp DNA Mini Kit (Qiagen, Germany, GmbH Catalogue no. 51304). Oligonucleotide primers supplied by Metabion (Germany) are listed in Table-2 [27-33].

**Table 2:** Description of primers and their respective amplicon size with cycling conditions.

Target gene	Primers sequences	Amplified segment (bp)	Primary denaturation	Amplification (35 cycles)			Final extension	Reference

				Secondary denaturation	Annealing	Extension		
*Listeria*								
*16S rRNA*	GGACCGGGGCTAATACCG AAT GATAA	1200	94°C	94°C	60°C /1 min	72°C/1 min	72°C/10 min.	[27]
	TTCATGTAGGCG AGT TGCAGCCTA		5 min	30 s				
*P. multocida*								
*Kmt1*	ATCCGCTATTTACCCAGTGG	460			55°C/1 min			[28]
	GCTGTAAACGAACTCGCCAC						
*Pseudomonas*								
*16S rRNA*	GGGGGATCTTCGGACCTCA	956		94°C/1 min	52°C/1 min			[29]
	TCCTTAGAGTGCCCACCCG							
*E. coli*								
*phoA*	CGATTCTGGAAATGGCAAAAG	720		94°C/30 s	55°C/45 s	72°C/45 s		[30]
	CGTGATCAGCGGTGACTATGAC						
*Staphylococci*								
*16S rRNA*	CCTATAAGACTGGGATAACTTCGGG	791			55°C/45 s			[31]
	CTTTGAGTTTCAACCTTGCGGTCG						
*S. aureus*								
*clfA*	GCAAAATCCAGCACAACAGGAAACGA	638			55°C/45 s			
	CTTGATCTCCAGCCATAATTGGTGG						
*C. pseudotuberculosis*								
*Pld*	ATA AGC GTA AGC AGG GAG CA	203			56°C/30 s	72°C/30 s		[32]
	ATC AGC GGT GAT TGT CTT CCA GG						
*Y. enterocolitica*								
*16S rRNA*	AAT ACC GCA TAA CGT CTT CG	330			62°C/45 s	72°C/45 s		[33]
	CTT CTT CTG CGA GTA ACG TC							

L. monocytogenes=Listeria monocytogenes, P. multocida=Pasteurella multocida, P. aeruginosa=Pseudomonas aeruginosa, Y. enterocolitica=Yersinia enterocolitica, C. pseudotuberculosis=Corynebacterium pseudotuberculosis, S. aureus=Staphylococcus aureus, E. coli=Escherichia coli

#### PCR amplification

Primers were used in a 25 μL reaction containing 12.5 μL EmeraldAmp Max PCR Master Mix (Emerald, Japan), 1 μL each primer of 20 pmol concentrations, 4.5 μL diethyl pyrocarbonate water, and 6 μL template. The reaction was performed in a Biometra T3 thermal cycler (Germany). Table-2 shows the thermal profile for each primer.

#### Analysis of the PCR products

PCR products were separated by electrophoresis according to Sambrook *et al*. [34]. Gelpilot 100 bp plus Ladders (11 bands) (Qiagen, Germany, GmbH) and Generuler 100 bp (10 bands) ladder (Fermentas, Germany) were used to determine fragment sizes. The gel was photographed using a gel documentation system (Biometra BDA digital, Germany) and the data were analyzed with the help of proprietary software (automatic image capture software protein simple formerly Cell Bioscience, USA).

## Results

After bacteriological examination of 200 different samples, 78 bacterial isolates were obtained. Serological testing of the isolates revealed seven different bacterial species of both Gram-positive and Gram-negative categories. The bacterial count was relatively high and ranged from 10^4^ to 10^7^ CFU/ mL. Using specific primers, all isolates were confirmed by the PCR technique (Table-3).

**Table 3:** Sources, numbers, and detection rates of samples from FTA cards after incubation for 24 h at 24-27°C.

Type of sample	No. of examined samples	Gram type	No. of +ve isolates	Isolates types	% of * +ve	Positive detection of viable cells onto FTA cards	

						No.	% **
Mastitis milk	33	G+ve	7	*S. aureus*	21.21	7	100
		G−ve	2	*P. aeruginosa*	6.06	1	50
Fecal samples	17	G−ve	5	*E. coli*	29.41	5	100
		G−ve	1	*Y. enterocolitica*	5.88	1	100
Skin lesions	4	G+ve	1	*C. pseudotuberculosis*	25	1	100
Raw milk samples	60	G+ve	5	*L. monocytogenes*	8.33	5	100
Internal organs from chickens (heart, spleen, liver, and lung)	86	G−ve	35	*E. coli*	45.3%	19	54.3
		G+ve	4	*S. aureus*	4.7%	4	100
		G−ve	18	*P. multocida*	20.9%	9	50
Total	200		78	------------------	39%	52	66.7

* Percent calculated according to the number of total samples’ type.

** Percent calculated according to the number of positive samples’ type. *L. monocytogenes=Listeria monocytogenes, P. multocida=Pasteurella multocida,* *P. aeruginosa=Pseudomonas aeruginosa, Y. enterocolitica=Yersinia enterocolitica, C. pseudotuberculosis=Corynebacterium pseudotuberculosis, S. aureus=Staphylococcus aureus, E. coli=Escherichia coli*

The effectiveness of FTA cards for detecting different species of bacteria by traditional bacteriological methods and purification of their nucleic acids was checked and As previously mentioned, four groups (A-D) of FTA cards were prepared and inoculated by the isolated bacteria. Inoculation was performed for all isolated bacterial cell liquid cultures and also impression smears from chicken organs for the first condition trial at 24 h 24-27°C.

In Group A, all Gram-positive bacteria (*S. aureus, Corynebacterium pseudotuberculosis*, and *L. monocytogenes*) were detected 24 h after inoculation at 24-27°C with detection percentage of 100%, while variations occurred in Gram-negative types on which the detection percentage from cards was 57.4%, even at very high cell concentrations. From Table-4, 19/35 of *E. coli*, 1/2 of *P. aeruginosa*, 1/1 of *Yersinia enterocolitica*, and 9/18 of *P. multocida* were detected. However, 20 positive randomly selected chicken internal organs (liver, spleen, heart, and lung impression smears) out of 32 were also inoculated onto cards and left at 24-27°C for 24 h, and all bacteria were detected from their selective agar plates and also confirmed by PCR.

**Table 4:** The detection results of inoculated FTA cards after 24 h at 24-27°C with respective to the Gram type.

Bacterial type	Total no.	Re-isolation from FTA cards (%)
Gram positive	17	17/17 (100%)
Gram negative	61	35/61 (57.4%)
Total	78	52/78 (66.7%)

The above-mentioned results revealed that the overall detection sensitivity of FTA cards of bacterial isolates was 66.7%, with 100% for detecting Gram-positive compared with Gram-negative related species.

All other FTA groups (B, C, and D) incubated at different conditions for different periods (4°C for 2 weeks, −20°C for 1 and 3 months, respectively) were also tested bacteriologically and no growth of bacteria was observed on the surface of bacterial selective agar media in all groups. We checked the availability of FTA cards for their efficacy of storage of bacterial live cells and for DNA detection at the previously mentioned storage conditions. The conventional PCR technique was used for amplifying target sequence of different conserved specific genes for each bacterial species using different sets of primers (Table-2).

**Figure-1:**
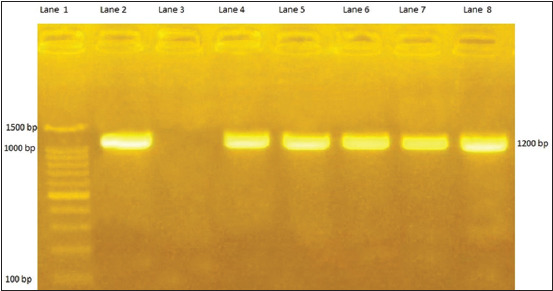
Agar gel electrophoresis for *Listeria monocytogenes*. The figure showing PCR products for the amplified 16 S RNA gene of *L. monocytogenes*. Lane 1 represents Gelpilot 100 bp plus ladder (11 bands). Lanes 2 and 3 represent positive and negative reference control. From lane 4 to lane 8 represent five *L. monocytogenes* strains at 1200 bp.

**Figure-2:**
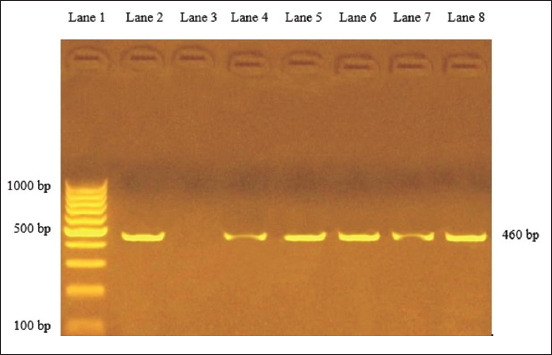
Agar gel electrophoresis for *Pasteurella* *multocida*. Lane 1 represents Gelpilot 100 bp ladder (10 bands). Lanes 2 and 3 represent positive and negative reference control for *P. multocida*, while from lane 4:8 represents positive amplification *P*. *multocida* strains at 460 bp.

All card groups (A, B, C, and D) nucleic acids showed their specificity to the examined gene primers. Positive amplicon of 16S ribosomal RNA gene (Figure-1) showed positive molecular weight of 1200 bp of five *L. monocytogenes* isolates. Also *P*. *multocida* species was confirmed by amplifying a specific fragment of the capsular gene (*kmt*1) that was amplified at 460 bp (Figure-2).

**Figure-3:**
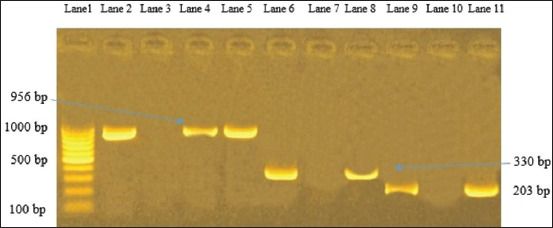
Agar gel electrophoresis for *Pseudomonas aeruginosa*, *Yersinia* *enterocolitica*, and *Corynebacterium pseudotuberculosis*. Lane 1 represents Generuler 100 bp ladder (10 bands), lanes 2 and 3 represent positive and negative reference control of *P. aeruginosa*. While lanes 4 and 5 represent positive amplification for *Pseudomonas* at 956 bp. Lanes 6 and 7 represent positive and negative control of *Y. enterocolitica*. While lane 8, the positive amplification of *Y. enterocolitica* at 330 bp. Lane 9 represents positive amplification for C. *pseudotuberculos*is at 203 bp. While lanes 10 and 11 negative and positive reference control for *Corynebacterium* spp.

**Figure-4:**
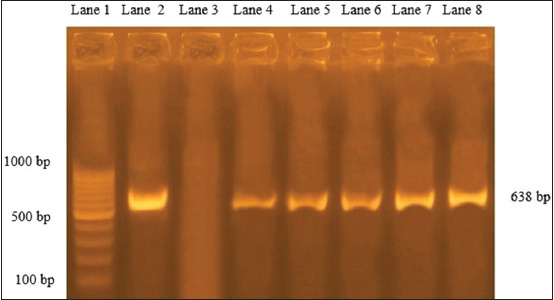
Agar gel electrophoresis for *Staphylococcus aureus*. Lane 1 represents Generuler 100 bp ladder (10 bands), lanes 2 and 3 represent positive and negative *S. aureus* reference control, lanes 4-8 represent positive *S. aureus* amplification at 638 bp.

**Figure-5:**
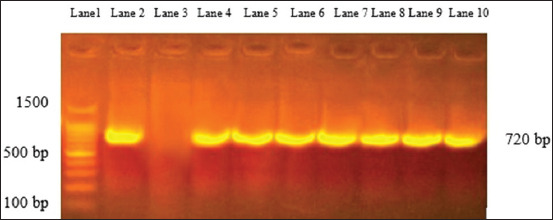
Agar gel electrophoresis for *Escherichia coli*. Lane 1 represents Generuler 100 bp ladder (11 bands), lanes 2 and 3 represent negative and positive reference of *E. coli*, lane 4:11 *E. coli*-positive amplification which appeared at 720 bp.

In Figure-3, 16S RNA gene for *Pseudomonas* *aeruginosa* and *Y*. *enterocolitica* is amplified and declared a specific molecular weight for the two species at 965 and 330 bp, respectively, and a positive fragment of 203 bp of phospholipase gene (*pld*) of *C*. *pseudotuberculosis* was moreover confirmed. Verifying the detection of *Staphylococcus* species from FTA cards by PCR 16 sRNA gene was amplified, moreover, another gene (clumping factor gene fragment *clfA)* for specifying *S*. *aureus* also showed positive amplification at 638 bp (Figure-4). In addition to the positive amplification of 720 bp of alkaline phosphatase gene (*phoA*), which is a common conserved gene of *E. coli* (Figure-5).

## Discussion

Fast and successful identification of microorganisms is a key point for diagnosis in the microbiology laboratory. Conventional identification is usually based on the phenotypic characters of organisms and it is laborious and time intensive. The technology of rapid kit method simplifies collection and storage of nucleic acids for molecular techniques and genetic analysis, thus reducing costs and time required [2]. Furthermore, the cards have been used to store different samples and for cytological specimens that should undergo various tests and molecular analyses [35,36] in addition to Green *et al*. [37], who also proved that the tissue for different organs is suitable to be placed onto cards and genetic profile can be evaluated.

The FTA method is cheap, rapid, and efficient for transporting bacterial DNA [37]. The structure of FTA cards and other commercial cards is paper-based system storing nucleic acids direct or contact to an organ [38]. This study discussed and evaluated the effectiveness of FTA commercial cards for transporting different samples to the laboratory as a vehicle and storage media.

FTA cards were used for collecting milk samples as a surveillance tool in cases of cattle mastitis as an easy tool for transportation and PCR detection [39]. Furthermore, they stated that the FTA technique simplifies DNA extraction and purification methods. In this study, as shown in Table-3, 200 different samples (milk samples, the skin lesion from diseased buffaloes, fecal samples, and chickens internal organs such as the lung, liver, heart, and spleen) were examined bacteriologically and the results were confirmed by conventional PCR (Figures-1-4). The examination revealed seven bacterial species such as *L. monocytogenes*, *E. coli*, *Y. enterocolitica*, *C. pseudotuberculosis, S. aureus*, *P. aeruginosa*, and *P. multocida* with bacterial counts ranging from 10^4^ to 10^7^ CFU mL. Furthermore, 78 bacterial cell cultures from both Gram types (Gram-positive and Gram-negative) were inoculated onto FTA cards and incubated for 24 h storage at 24-27°C. It revealed that the Gram-positive bacteria were detected from cards with 100%, while that of Gram-negative detection were 57.4% (Table-4). Nearly similar results were obtained by Rajendram *et al*. [1], recording the viability of bacterial cells retained on the FTA cards varied among broad groups of bacteria, where the more fragile Gram-negative species, no viable cells were retained even at high cell densities 10^7^ and 10^8^ CFU mL^−1^, while the Gram-positive species showed viable cell growth. Furthermore, the difference between Gram-positive and Gram-negative was measured by acids [40] as the difference may occur due to the intact cell with healthy wall as Gram-positive bacteria cytoplasmic membrane is covered by a thick (20-80 nm) cell wall layer comprising thick peptidoglycan layers with murine and other components like polysaccharides. In addition, several bacteria of Gram-positive types have additional secondary cell wall structure called polymers, having a significant role in bacterial cell surface attachment [41].

When 20 chicken organs impression smears were selected randomly and examined on FTA cards, it showed specific characters of colonies for the isolated bacteria with confirmation of relevant nucleic acids at 24-27°C. These results nearly agree with that mentioned in Green *et al*. [37], in which the cards were valid for transporting tissue samples and tissue impression smears from inner organs, which is appropriate to put on FTA cards.

Assessment for preserving DNA from swabs onto FTA cards in different environments has been simple for both field and laboratory operation at various conditions [42]. The cards have a critical role in saving time, especially in some epidemics to minimize the requirement of culturing methods and long-distance transportation [43]. Our results confirmed the previous findings regarding detecting bacterial DNA from FTA cards as all cards were positive for DNA detection using conventional PCR in all mentioned conditions, which agreed with the results obtained by Rajendram *et al*. [1] who tested 100 samples stored on FTA cards at ambient temperature and were successfully purified by DNA sequencing using the target 16S RNA gene.

As all bacterial DNA have been confirmed, and the results matched with that of Almeida *et al*. [19], who detected *P. multocida* DNA in 100% of the samples and concluded that the FTA cards can be used for the storage period up to 35 days at different temperatures (4°C and 37°C).

Accordingly, the study of Jóźwiak *et al* [44] proved that the long term storage at −20°C enhances the better stability for detection in comparison to 24-27°C. Also the usage of cards greatly limit the possibility of infection and gave a long term nucleic acid storage. Similar conclusion was stated by FTA card manufacturer (Whatman Plc., UK) that the card could be used for sampling and transportation of even sensitive RNA viruses and it can be stored for a short time at 24-27°C or may be a longer time at −20°C and deep freezing −70°C [45], and no difference in sensitivity of FTA cards during the detection of nucleic acid under different conditions; this conclusion validated similar assumption obtained by Rabodoarivelo *et al*. [46] who evaluated the sensitivity and specificity of FTA cards for detection of selected strains of some types of tuberculosis bacteria and stated that method was of good for sensitivity and specificity. Furthermore, the stability of DNA on cards has been documented during the examination of different surgical specimens and stored at 24-27°C [47].

The genetic material (DNA) was safely fixed onto cards and protected from damage and degradation, making high-quality diagnosis and analysis by different molecular techniques [14]. It is possible to recover sufficient amounts of DNA for human identification, even after more than a decade of storage at ambient temperature [8].

The long-term DNA storage with high fidelity at various temperatures makes the FTA cards a good alternative way for gathering samples and suggested a way for the suitability of storing and transporting nucleic acid profitably for further molecular application [19]. Moreover, the demonstration of the FTA cards of overall agreement in detecting HPV level and genotyping when compared with other transport media also showed high-quality nucleic acid required for testing the molecular technique when use different methods to describe the nucleic acid from different samples and tissues by different methods for downstream molecular applications [48]. The constancy for DNA storage onto FTA cards was measured by Chandrashekara *et al*. [49] and confirmed that DNA was stable up to 6 months.

In addition to the overall conclusion of Sarangi *et al*. [50] about the advantages of FTA^®,^suggesting that FTA card holds promise as an alternative system for the transportation of organs harbor an infectious agent (viruses and bacteria) concerning different storage conditions, different assays.

Our study confirmed using FTA in transporting tissue samples and culture of bacteria for phenotypic characterization, especially of public health concern and other bacteria of food health problems for only 24 h. However, concerning using FTA card for molecular applications, it is valid for longer periods even at different temperatures. The technique requires further studies for many bacterial species.

## Conclusion

We evaluated FTA card efficacy as a tool for storage of bacterial live cells of animal origin and their DNA over a period of approximately 3 months; also, we checked the safe transport of those bacterial agents. We clarified that it is possible to transport live bacteria of Gram-positive type, especially if the source is from organ impression smears using this method but not exceeding 24 h at 24-27°C. However, the bacterial nucleic acid can be safely detected at 3 months.

## Authors’ Contributions

AGS designed the study, performed the molecular experiments, and drafted the manuscript. NRB participated in the conception and design of the study. AAEM contributed in samples collection and bacteriological examination. AAK Contributed in acquisition of data, analysis and interpretation of the results, and critical reviewing. All the authors have approved the final article version to be submitted.
